# Experimental analysis of enhanced finite set model predictive control and direct torque control in SRM drives for torque ripple reduction

**DOI:** 10.1038/s41598-024-65202-1

**Published:** 2024-07-22

**Authors:** Deepak M, Devakirubakaran Samithas, Praveen Kumar Balachandran, Shitharth Selvarajan

**Affiliations:** 1 Department of Electrical and Electronics Engineering, KIT-Kalaignarkarunanidhi Institute of Technology, Kannampalayam Post, Coimbatore, Tamil Nadu 641402 India; 2 Center for Nonlinear Systems, Chennai Institute of Technology, Kundrathur, Chennai, Tamil Nadu 600069 India; 3 Department of Electrical and Electronics Engineering, Vardhaman College of Engineering, Hyderabad, 501218 India; 4 Department of Electrical, Electronic and Systems, Faculty of Engineering and Built Environment, Universiti Kebangsaan Malaysia, Selangor, Malaysia; 5 Department of Computer Science and Engineering, Kebri Dehar University, Kebri Dehar, Ethiopia

**Keywords:** Switched reluctance motor, Direct torque control, Finite set model predictive control, Dynamic modelling, Electrical and electronic engineering, Mechanical engineering

## Abstract

The magnet-less switched reluctance motor (SRM) speed-torque characteristics are ideally suited for traction motor drive characteristics and its advantage to minimize the overall cost of on-road EVs. The main drawbacks are torque and flux ripple, which have produced high in low-speed operation. However, the emerging direct torque control (DTC) operated magnitude flux and torque estimation with voltage vectors (VVs) gives high torque ripples due to the selection of effective switching states and sector partition accuracy. On the other hand, the existing model predictive control (MPC) with multiple objective and optimization weighting factors produces high torque ripples due to the system dynamics and constraints. Therefore, existing DTC and MPC can result in high torque ripples. This paper proposed a finite set (FS)-MPC with a single cost function objective without weighting factor: the predicted torque considered to evaluate VVs to minimize the ripples further. The selected optimal VV minimizes the SRM drive torque and flux ripples in steady and dynamic state behaviour. The classical DTC and proposed model were developed, and simulation results were verified using MATLAB/Simulink. The proposed model operated in SRM drives experimental results to prove the effective minimization of torque and flux ripples compared to the existing DTC.

## Introduction

The growth of electric vehicles demands low cost and high mileage in future. The researchers are interested in reducing the EV subsystem costs, such as electrical machines and batteries^1,2^. Currently using limited resources, permanent magnet machine materials such as neodymium, and samarium come with a high cost^3^. Materials are preferred for their high torque/power density and high efficiency. However, PM-based EVs have a demagnetization effect, fault-tolerant issues, and rare earth material increasing cost rapidly^4^. The researcher's attention towards low-cost silicon material switched reluctance motor characterises perfectly suit to EVs power/torque base speed drive^5,6^. Compared to other polyphase machines, it gains attention for several applications because of its simple construction, robustness, high power density, low cost, long constant power range, and enhanced reliability for fault-tolerant capability^7^. However, the main issues are controllability, torque density, torque ripple, vibration, and acoustic noise. To improve the above problems, many research articles, reviews, and literature surveys have reported individual performance parameters and multiobjectives in earlier works^8–10^. These issues are mainly mitigated by changing the motor geometry optimal design and power electronics converter control drive. In^11^ design aspects, high-quality steel and slot fill factor in double salient pole SRM improves the efficiency by 6% and generates high torque ripple. In^12–16^, many researchers used segmented SRM topology, which has a simple construction, compact size, fewer losses, and high flux linkage per turn has increased the output torque with single tooth winding topology compared to full pitch winding. There are many researchers continuously improving the performance of high-potential SRM technology for EVs. In^17,18^, the new topology double stator SRM has an improved torque/weight ratio, minimizing vibration and noise, but it generates a high torque ripple. Many researchers further improve the performance of SRM design in all aspects of EVs. An increasing number of phases has been reducing the torque ripple^19^. The axial flux SRM reduces the torque ripple compared to radial flux, but the complexity of design and cost is high^13^. A power electronic converter chosen based on the stator phase has been proposed a conventional half-bridge converter has high fault-tolerant capability, reduces ripples, and uses a smaller number of components. Modern finite element analysis and optimization tools are effectively used to reduce the torque ripple^20,21^. However, compared to design topology, the power electronics controller is used in SRM to mitigate torque ripple, acoustic noise, and vibration and address their challenges in recent research articles.

In the literature of recent years^22,36^, SRM has developed many of its drawbacks have been overcome by recent advancements in the design and non-linear control strategy. In^23,24,35^, torque sharing function (TSF) and direct torque control (DTC) methods are adopted to control the torque and dynamic response, but they are incapable of torque ripple minimization. In^24^, the selection of eight sector partitions and effective VVs is minimal, which can result in high torque ripple and good dynamic response. The DTC method is a well-established control principle for minimizing torque ripple effectively. The DTC has successfully controlled the objectives of linear characteristics of AC machines and balanced three-phase sinusoidal excitation. The conventional DTC method uses a two-hysteresis magnitude flux linkage and torque loop^24^ by a space vector voltage and selects an improper switching state to produce more torque ripples. In^25^, the selection of hysteresis loop width for flux and torque is within the limit, which may cause improper switching VVs to produce high torque ripple. In^26^, the DTC sector partition for SRM varies depending on the phase, increasing and decreasing sector changes the switching look-up tables affect the change in speed acceleration and deacceleration produce more torque spikes and ripples. On the other hand^27^, MPC has attention to SRM drive advantages: improved performance, flexibility, adaptability, energy efficiency, fast dynamic response, fault-tolerant operation, and reduced system complexity, owing to its simple principle, ease of understanding, and online optimization. In^28,29^ literature survey, different advanced MPC control techniques are proposed: finite control set (FCS)-MPC, the optimum duty ratio calculation-MPC, explicit-MPC, maximum torque per ampere-MPC, direct-MPC, continuous control set MPC, adaptive-MPC and weighting less-MPC. In^28^, a predictive current control technique for applications requiring low torque ripple and low acoustic noise is proposed. In^29^, a model predictive current control of SRM that considers inductance variations and measurement uncertainties is proposed. To reduce torque ripple, copper loss, and average switching frequency, a model predictive torque control for low-speed operation of SRM (constant torque region) is proposed in^30^. The impact of the number of prediction horizons on FCS-MPC's ability to control low-speed torque is proposed^31^. In conventional hysteresis current MPC^32^, reference and phase current do not reach constant value at dynamic behaviour, producing high error around the unaligned position. Also, it is unable to operate in the four quadrants due to back-EMF. In^33^, MPC is designed in four-phase SRM to control the objective constraints individually, and it is not easy to optimize the weighting factor. In^34^, using the weighted cost function brings flexibility to control multiple objectives. However, from the literature article, both conventional DTC and MPC techniques have few drawbacks to calculating self-inductance, sector partition, voltage vector selection, prediction horizon, selection of control objectives cost function and few function block transformations resulting in high flux and torque ripple.

To overcome the problems mentioned, this paper proposes existing FS-MPC-based novel VV selection that includes single cost function objectives with a single horizon of current and torque prediction for four-phase SRM. The MPC selects optimal VV switching and is directly applied at each sampling time. The highlights of the proposed FS-MPC Model are given as,

- Develop a mathematical model that accurately represents the dynamics of the 8/6 SRM.
- The DTC scheme focuses on the modelling of motor behaviour, sector partitioning, and selection of VVs switching state.
- In the MPC model, a mathematical model to estimate phase current and rotor position, prediction of torque at the single horizon, cost function control objectives, and selection of VVs switching state.
- An enhanced FS-MPC is developed with minimum cost function formulation, prediction horizon, control horizon, constraints considerations, control algorithm, and output estimation to improve control performance.
- A single cost function minimizes the error to obtain an optimal active voltage vector that selects the switching state.
- The proposed control algorithm is simple and compared with other control techniques.

**Figure 1:**
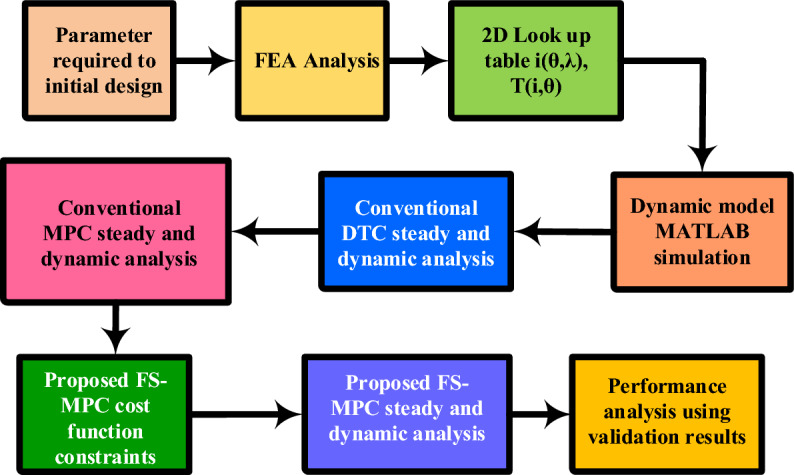
Organization flow.

This paper is organized as follows, depicted in Fig. 1: Section “Modelling of SRM” includes the mathematical modelling for four-phase SRM for low-power EVs. Section “Classical control techniques” discusses the problem associated with conventional control techniques such as direct torque control, and model predictive control, including the performance of torque and flux ripple. The proposed FS-model predictive control techniques are discussed in section “Proposed finite SET-MPC”. The simulation and experimental hardware setup results and validation performance analysis are compared with the existing model in section “Result and discussion”. The final section is the conclusion.

## Modelling of SRM

### Dynamic modelling

A 2.2 kW, four-phase 8/6 SRM is designed and analysed with the magnetic characteristics using MAGNET infolytica software. The relationship between motor variables such as flux linkage, phase current and rotor position are captured to develop the look-up table (LUT), and their corresponding inputs (e.g., voltage and phase current). The double pole SRM alignment of the rotor poles with the maximum and minimum inductance values is a critical aspect that affects the motor operation and performance.

**Figure 2:**
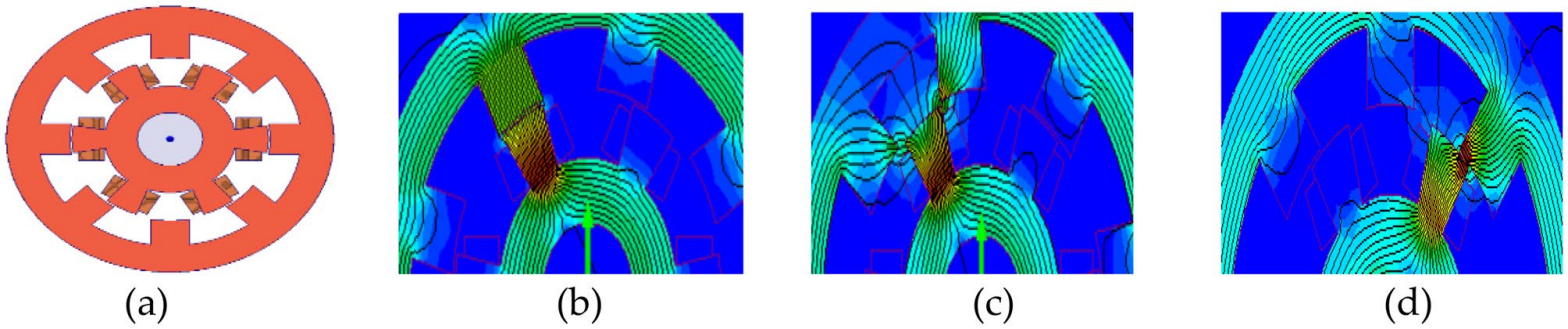
SRM electromagnetic analysis (**a**) 8/6 (**b**) Aligned (**c**) Unaligned (**d**) Middle aligned.

**Figure 3:**
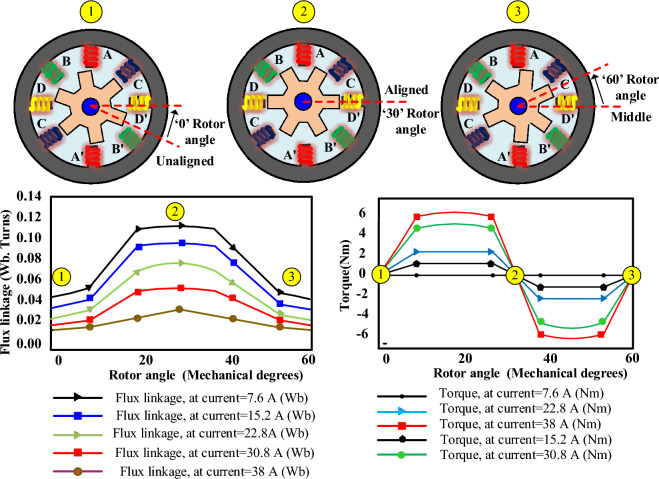
8/6 SRM, Current vs (flux linkage, rotor position), Torque vs (current, rotor position).

Figure 2 shows unalignment, middle alignment, and alignment between the stator and rotor poles of SRM, respectively. Understanding and controlling the alignment states of an SRM can help in achieving efficient torque production, reducing losses, and enhancing overall motor performance. Hence, the degree of inductance distortion at different positions is nonlinear. Therefore, an inductance curve can be divided into eight regions by the intersections of four phase inductances, and each region is 45°. The effect of magnetization characteristics on the phase inductance distribution with flux linkage and rotor position is analysed by FEA power at 2.2 kW four phase 8/6 pole SRM, as shown in Fig. 3.

The mathematical representation of a double pole SRM neglecting mutual inductance between poles can be expressed using the single-phase voltage equation is

1 $$v = i_{p} r_{s} + \frac{{d\psi_{p} (i_{p} ,\theta )}}{dt}$$

2 $$v = i_{p} r_{s} + \frac{{\partial \psi_{p} }}{\partial \theta } \cdot \frac{d\theta }{{dt}} + \frac{{\partial \psi_{p} }}{{\partial i_{p} }} \cdot \frac{{di_{p} }}{dt}$$

where *v* is phase voltage,* i*_*p*_ is phase current, *r*_*s*_* is* stator resistance, *ψ*_*p*_ = phase flux linkage, and *ө* is rotor position, it changes the flux linkage maximum at pole alignment and minimum in unaligned flux linkage.

Based on the derivation of the magnetic co-energy, the phase torque can be described as

3 $$T_{p} = \frac{{\partial W_{co} (i_{p} ,\theta )}}{\partial \theta }$$

The mathematical model developing from the flux linkage, rotor position, and phase current is required to identify the non-linear inductance profile. The LUT is developed when different phase currents change to identify the saturated and unsaturated flux linkage with rotor position *i*(*ө, ψ*) electromagnetic torque changes due to stator phase current with rotor position *T *(*ө, i*)*. *The magnetization characteristics of flux linkage at different rotor positions and torque at different rotor positions are used to develop a 2D-LUT build analytical model.

Let, the dynamics of SRM four-phase current be described by a differential equation using (1), which can be rearranged

4 $$\frac{{di_{p} }}{dt} = \frac{1}{{\frac{{\partial \psi_{p} }}{{\partial i_{p} }}}}\left( {v - r_{s} i_{p} - \frac{{\partial \psi_{p} }}{\partial \theta }\omega } \right),\quad p = 1,2,3,4$$

where, *p* is the number of phases, $\frac{{\partial \psi_{p} }}{\partial i}$ is a partial derivative of flux linkage with respect to phase current and $\frac{{\partial \psi_{p} }}{{\partial \theta_{p} }}$ is the partial derivative of flux linkage with respect to rotor position.

Where *T* is electromagnetic torque, *T*_*ph*_ is phase torque in an SRM based on the magnetic energy variation.

The load torque equation of SRM $\frac{d\omega }{{dt}}$ is the rate of change of angular velocity expressed as

5 $$\frac{d\omega }{{dt}} = \frac{1}{j}\left[ {T_{e} - T_{L} - B\omega } \right]$$

where *T*_*L*_ is load torque, *B* is damping coefficient, and *j* is moment of inertia.

The analytical model and measurement of SRM parameters that can be described with sufficient accuracy are listed in Table 1. Equations (1) to (4) are used to develop the mathematical model in MATLAB/Simulink platform for SRM parameters are listed in Table 1.

**Table 1:** SRM dynamic model specification.

Parameter	Values
Stator resistance	1.2 Ω
Inertia	0.0089 kg.m.m
Friction	0.01 N.m.s
Flux linkage	0.584 Wb
Aligned inductance	4 mH
Unaligned inductance	0.55 mH
Saturated aligned inductance	2.37 mH
DC link voltage	60 V
Rated power	2.2 Kw
Rated Speed	1200 rpm
Stator diameter	210 mm
Stack length	121 mm
Airgap	0.4 mm

### Asymmetric half-bridge converter (AHBC)

**Figure 4:**
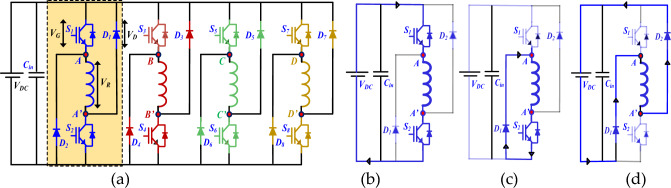
SRM (**a**) 4-phase AHBC (**b**) Magnetization '1' (**c**) Freewheeling '0' (**d**) Demagnetization '− 1’.

The 8/6 SRM is driven by an efficient power converter module four-phase asymmetric half-bridge converter (AHBC) circuit, as shown in Fig. 4a. Each phase is conducted individually at unidirectional power flow. The switching states for four phases (*S*_1_,* S*_4_), (*S*_3_,* S*_6_), (*S*_5_,* S*_2_) and (*S*_7_,* S*_8_).

The phase voltages can be divided into three levels: magnetization (*V*_*dc*_*,* =  + 1), freewheeling (*V*_*dc*_ = 0), and demagnetization (*V*_*dc*_*,* = − 1), as shown in Fig. 4b–d, respectively. Therefore, the per-phase winding can be energised and provide the three different level output switching states listed in Table 2. In this case, the number of phases increases at the same time, and switching state levels also increase with higher sampling time. In the basic three-phase converter, each phase circuit selected (*3*^*3*^ = 27) different switching states at each sampling time. The four and five-phase switching state levels are *3*^4^ = 81 and *3*^*5*^ = 243, respectively. The optimal voltage vector level selected for three phases is from these twenty-seven switching states in a sampling interval of a single prediction horizon. The length of the prediction horizon increases in three phases (27^2^ = 729), and the computational burden time increases exponentially. For example, prediction length increases two times in the horizon for four and five phases (81^2^ = 6561) and (243^2^ = 59,049), respectively.

**Table 2:** Mode of operation AHBC single phase.

Mode of operation	S_1_	S_2_	D_1_	D_2_
Magnetization	1	1	0	0
Freewheeling mode	1	0	1	0
Freewheeling mode	0	1	0	1
Demagnetization mode	0	0	1	1

The analytical model in MATLAB/simulation to implement different control techniques in SRM to analyse the torque and flux ripple. Therefore, the advanced control techniques such as direct torque control, model predictive control and proposed enhanced finite control set- MPC are discussed in the section.

## Classical control techniques

### Direct torque control (DTC)

**Figure 5:**
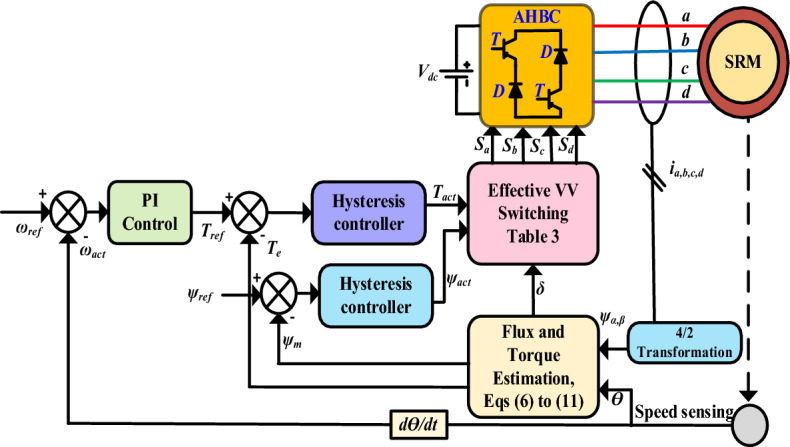
Conventional DTC method.

The classical DTC functional block diagram is shown in Fig. 5. The direct torque control using two hysteresis controllers flux magnitude (*ψ*) and torque (*T*_*e*_) in the input of the switching table selects effective VVs (*S*_*a*_*, S*_*b*_*, S*_*c*_, and *S*_*d*_). The SRM rotor position and phase current or voltage are used to derive the flux magnitude, angle, and torque estimation for DTC techniques. Therefore, the hysteresis control band maintain two variables set as maximum and minimum limits. DTC principle selects the optimal voltage vector for the corresponding flux magnitude and torque position. In four-phase SRM, 81 effective VVs and 17 active VVs. The voltage can be calculated from (1) a modified single-phase flux linkage equation expressed as

6 $$\psi_{s} = \int\limits_{0}^{t} {(v - R_{s} i)} + \psi_{i}$$

where *ψ*_*i*_ is the initial stator flux in the phase.

The estimation of torque written from (3) using a block diagram can be expressed as

7 $$\begin{gathered} T(\theta ,i) = i\frac{\partial \psi (\theta ,i)}{{\partial \theta }} \hfill \\ \hfill \\ \end{gathered}$$

where, *ө* = rotor position angle, *i* = phase current.

**Figure 6:**
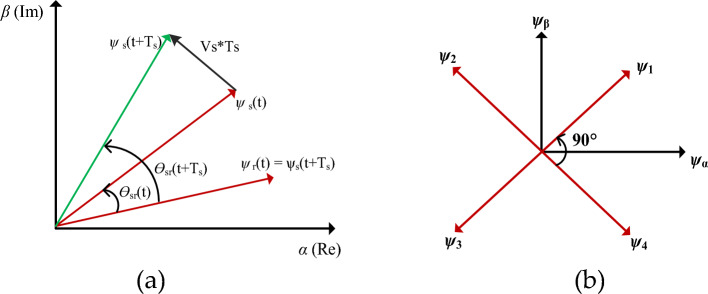
DTC principle (**a**) Flux and torque estimation (**b**) Four phase VV at 45°.

**Figure 7:**
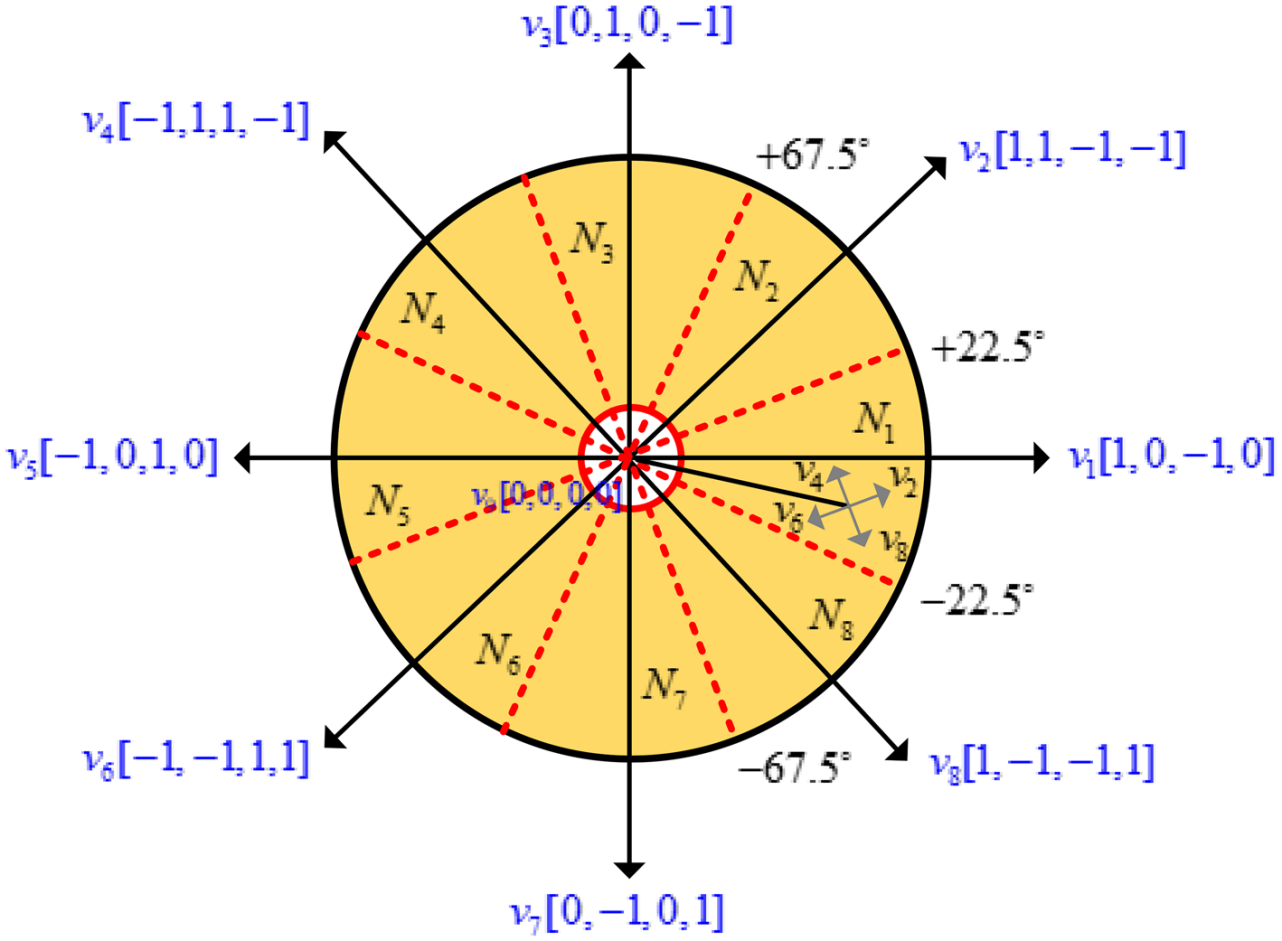
Four-phase SRM adopted DTC active voltage vectors.

The principle of the DTC scheme for flux magnitude and torque position for one sector is shown in Fig. 6a. The double salient SRM, voltage vector for each phase winding is derived at a 90° angle on the centre axis of the stator pole, as shown in Fig. 6b. The DTC for four-phase SRM has an equal magnitude of eight active VV and eight sectors, as shown in Fig. 7.

The active VVs are (*V*_*1*_, *V*_*2*_, *V*_*3*_, *V*_*4*_, *V*_*5*_, *V*_*6*_, *V*_*7*_ and *V*_*8*_) at the angle of 45°. The number of sectors is chosen eight (*N*_*1*_, *N*_*2*_, *N*_*3*_, *N*_*4*_, *N*_*5*_, *N*_*6*_, *N*_*7*_ and *N*_*8*_ at interval of each vector is (22.5° < *V*_*1*_ < − 22.5°), respectively. The stator flux vectors of the four phases of SRM are transformed into stationary orthogonal two-axis reference ($I_{\alpha ,} I_{\beta }$) transformation to derive the actual flux vector. The phase VVs single axis is transferred to orthogonal components in $(\psi_{\alpha } )$ and $(\psi_{\beta } )$.

8 $$\psi_{\alpha } = \psi_{1} \cos 45^{^\circ } - \psi_{2} \cos 45^{^\circ } - \psi_{3} \cos 45^{^\circ } + \psi_{4} \cos 45^{^\circ }$$

9 $$\psi_{\beta } = \psi_{1} \sin 45^{ \circ } + \psi_{2} \sin 45^{ \circ } - \psi_{3} \sin 45^{ \circ } - \psi_{4} \sin 45^{ \circ }$$

The equivalent flux vector can be calculated by flux magnitude $(\psi_{m} )$ and angle $(\partial )$,

10 $$\psi_{m} = \sqrt {(\psi_{\alpha } )^{2} + (\psi_{\beta } )^{2} }$$

11 $$\partial = arctg\left( {\frac{{\psi_{\beta } }}{{\psi_{\alpha } }}} \right)$$

**Figure 8:**
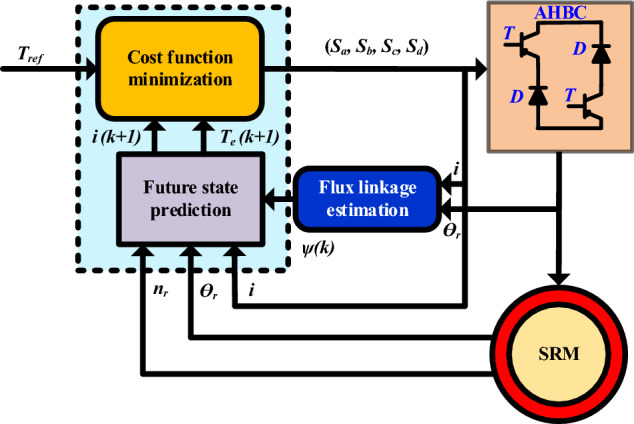
Conventional MPC for SRM.

The hysteresis controller maintains torque and flux control within a band by knowing the flux magnitude and torque. The sector partition is divided by 45° of each voltage sector, for *V*_*1*_ sectors (22.5° < *V*_*1*_ < − 22.5°). Similarly, other voltage vectors are partitioned as depicted in Fig. 8. If the estimated flux in sector 1 is placed, the increasing flux ($\psi_{m} = 1$) and increasing torque (*T*_*act*_ = 1*)* select the VVs as *V*_*6*._ Similarly, the selection of VVs depends on increasing or decreasing torque and flux linkages. The switching tables are listed in Table 3, respectively. This model is developed in MATLAB/Simulink platform and simulation results for static and dynamic speed response, as discussed in the result section.

**Table 3:** DTC Switching table^32^.

N (sectors)	1	2	3	4	5	6	7	8
$$\psi_{act} = 1$$								
* T*_*act*_ = *1*	*V*_*6*_	*V*_*7*_	*V*_*8*_	*V*_*1*_	*V*_*2*_	*V*_*3*_	*V*_*4*_	*V*_*5*_
* T*_*act*_ = *0*	*V*_*8*_	*V*_*1*_	*V*_*2*_	*V*_*3*_	*V*_*4*_	*V*_*5*_	*V*_*6*_	*V*_*7*_
$$\psi_{act} = 0$$								
* T*_*act*_ = *0*	*V*_*2*_	*V*_*3*_	*V*_*4*_	*V*_*5*_	*V*_*6*_	*V*_*7*_	*V*_*8*_	*V*_*1*_
* T*_*act*_ = *1*	*V*_*4*_	*V*_*5*_	*V*_*6*_	*V*_*7*_	*V*_*8*_	*V*_*1*_	*V*_*2*_	*V*_*3*_

$\psi_{act}$ = Flux linkage, T_act=_ Torque, 1 = Increasing, and 0 = Decreasing.

### Model predictive control (MPC)

The classical MPC functional block diagram is shown in Fig. 8. The phase current (*i*), and rotor position (*ө*_*r*_) are used to calculate an estimation of flux linkage *λ(k)*, torque *T*_*ph*_*(k)* and current *i(k)*. The four-phase current transformed two orthogonal reference frames to predict the future step response of *T*_*e*_*(k* + 1) and *i(k* + 1)*.* The torque and current prediction for future step input (*k* + *1*) of the cost function MPC. The summing of actual and reference speed error given to proportional integral control output produces reference torque (*T*_*ref*_), respectively.

The Eq. (1) can be modified to a discrete model using the Euler method is

12 $$v(k) = Ri(k) + \frac{\psi (k + 1) - \psi (k)}{{T_{s} }}$$

*T*_*s*_ = sampling time interval, *k* = time instant, and ψ(*k* + *1*) = flux linkage for SRM one future time step is derived using

13 $$\psi (k + 1) = \psi (k) + T_{s} (v(k) - Ri(k))$$

The flux linkage of one future time step function is based on current phase flux linkage, phase current, phase voltage, and sampling time interval of the machine.

Flux linkage of the current position depends on both rotor position and phase current feedback.

14 $$\psi (k) = \psi (i(k),\theta_{mech} (k))$$

The flux linkage of the future step is predicted by the current state flux linkage.

The time constant of SRM mechanical behaviour is much higher than the electrical behaviour, at any particular time period, and speed is assumed to be constant. Hence, the one-step mechanical rotor position (*k* + 1) calculated

15 $$\theta_{mech} (k + 1) = \theta_{mech} (k) + \frac{360}{{60}}n_{r} T_{s}$$

*n*_*r*_ = rotor speed, once the one-step flux linkage and rotor position are, the phase current for future step (*k* + 1) is calculated

16 $$i(k + 1) = i(\psi (k + 1),\theta_{mech} (k + 1))$$

From (11) to (16) used to calculate the phase torque

17 $$T_{phase} (k + 1) = T_{phase} (i(k + 1),\theta_{mech} (k + 1))$$

The total phase torque can be calculated based on the number of phases used in the SRM converter

18 $$T_{e} (k + 1) = \sum\limits_{m = 1}^{j} {T_{j} } (k + 1)$$

where, *j* = number of phases, the SRM future state can be found from Eqs. (11) to (18). The unknown variable is phase voltage *v(k)* determined effective 81 VVs to 9 active VVs. This helps the control algorithm and hardware implementation become less complex, reducing computational burden time. Additionally, it is important in real-time control systems where quick and efficient computations are necessary.

19 $$\min K(VV_{{s_{a} ,s_{b} ,s_{c} ,s_{d} }} ) = (T_{e} (k + 1) - T_{ref} )^{2} + k_{1} \sum\limits_{m = 1}^{4} {i_{k + 1}^{2} }$$

where, *k*_*1*_ is the weighting factor, *K* is the cost function, and four-phase switching states (*S*_*a*_*, S*_*b*_*, S*_*c*_*, and S*_*d*_) are selected by optimal active VVs (V_*1*_, V_*2*_, V_*3*_,…, V_8_). However, the cost function (19) shows the total torque prediction. This cost function considers two control objectives: provide high torque ripple, system control complexity and reduce copper loss reduction correlated to the square of the current. This classical MPC-SRM drive is implemented in MATLAB/ Simulink.

## Proposed finite SET-MPC

**Figure 9:**
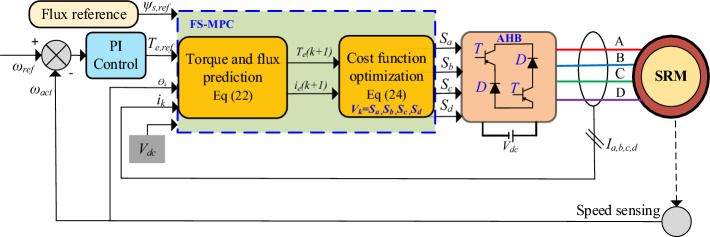
Proposed FS-MPC scheme for SRM.

The proposed finite set (FS)-MPC applied to AHBC-fed SRM drive is presented in Fig. 9 which involves four-phase SRM, AHBC, future state prediction (torque prediction), rotor position, and current detection functional block diagram. The classical AHBC voltage vector switching signals (*V*_*k*_) = [*S*_*a*_*, S*_*b*_*, S*_*c*_*,* and *S*_*d*_] is applied to represent the switching states of the AHBC for the four-phase SRM. At first, the rotor position (*ө*_*k*_) and phase current (*i*_*k*_) are detected for sector partition to select VVs using flux prediction. The phase flux, torque, and current at the period (k + 1) that is predicted by estimating from phase current and rotor position at the kth step. The optimal VVs are selected by minimizing cost function control objectives. The input of the cost function is reference torque (Te, ref), and current (i.e., ref) is obtained from speed and current deviation through the PI controller.

### Cost function and constraint estimation

#### Voltage vector selection

The topology of the AHB converter has three modes of states for each phase (1, 0, − 1). A DC link voltage (*V*_*DC*_) is applied in the four-phase winding can be expressed as

20 $$\begin{gathered} {\text{Magnetization}} = {1},\quad V_{p,1} = V_{DC} - 2V_{G} - V_{R,L} \hfill \\ {\text{Free wheeling}} = 0,\quad V_{p,0} = - (V_{D} + V_{G} + V_{R,L} ) \hfill \\ {\text{Demagnetization}} = - {1},\quad V_{p, - 1} = - (V_{DC} + 2V_{D} + V_{R,L} ) \hfill \\ \end{gathered}$$

where, *V*_*DC*_*, V*_*G*_*, V*_*D*_, and *V*_*R*_ are DC link voltage, IGBT voltage drop, Diode voltage drop, and resistance voltage drop, respectively.

**Figure 10:**
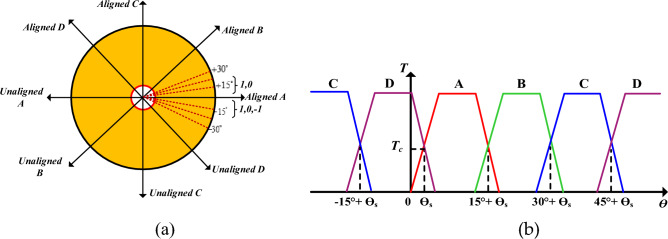
SRM (**a**) VV sector rotor angle (**b**)Torque contributions.

The four-phase AHB switching state is *3*^4^ = 81 VVs. However, 81 VVs applied to torque prediction of four phases SRM selected under any condition, which increases the burden of CPU and the execution time of the algorithm takes much time. Hence, it is necessary to delete some unreasonable four-phase basic voltage states, reduce the execution time, and reduce the total number of effective VVs can be decreased. Therefore, the proposed MPC uses effective VVs state 81 to 8 active VV state as shown in Fig. 10. Consider 8/6 SRM as an example; the conduction sequence is *A-B-C-D*, respectively. The mechanical angular period is 60°.

Therefore, the proposed FS-MPC new switching table is listed in Table 4. The improved switching table for 8/6 SRM, where ө1 represents the position of the stator pole overlaps the rotor pole as shown in Fig. 6. Table 4 represents the eight sectors (*N*_*1*_ to *N*_*8*_) with example in sector 1, the position from 0 to ө1 in Phase A, which is located unaligned position and derivative of the inductance is very low. In this condition, the switch state is set as “0” and “1”. In the same region, the derivative of the inductance for Phase D is very high, and positive torque is produced in this Phase; thus, the switch state is set as “1”, “0”, and “− 1”. Hence, Phase B and C are demagnetized “− 1”. Similarly, each sector's derivative inductance, aligned position, and demagnetization of phases are listed in Table. 4.

**Table 4:** Selection of four-phase proposed switching table.

Switching state	Position $\left[ {\theta_{A} } \right]$	Phase A (*S*_*a*_)	Phase B (*S*_*b*_)	Phase C (*S*_*c*_)	Phase D (*S*_*d*_)
Sector 1	$\left[ {0^\circ ,\theta_{1} } \right]$	1,0	− 1	− 1	1,0, − 1
Sector 2	$\left[ {\theta_{1} ,15^\circ } \right]$	1,0, − 1	− 1	− 1	0, − 1
Sector 3	$\left[ {15^\circ ,15^\circ + \theta_{1} } \right]$	1,0,− 1	1,0	− 1	− 1
Sector 4	$\left[ {15^\circ + \theta_{1} ,30^\circ } \right]$	0, − 1	1,0, − 1	− 1	− 1
Sector 5	$\left[ {30^\circ ,30^\circ + \theta_{1} } \right]$	− 1	1,0, − 1	1,0	− 1
Sector 6	$\left[ {30^\circ + \theta_{1} ,45^\circ } \right]$	− 1	0, − 1	1,0, − 1	− 1
Sector 7	$\left[ {45^\circ ,45^\circ + \theta_{1} } \right]$	− 1	− 1	1,0, − 1	1,0
Sector 8	$\left[ {45^\circ + \theta_{1} ,60^\circ } \right]$	− 1	− 1	0, − 1	1,0, − 1

### Torque estimator and cost function

The objective function of MPC selection based on instantaneous torque and current with reference values minimizes the error.

**Figure 11:**
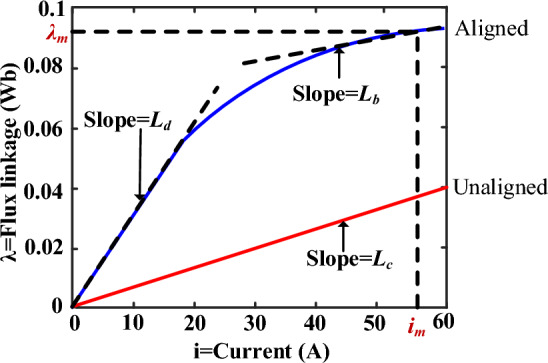
Flux linkage characteristics.

As for the researched 8/6 SRM, the magnetization curve at aligned and unaligned positions is shown in Fig. 11, where relevant parameters in the above formulas are marked. On basis of this, we obtain N_r_ = 8, L_d_ = 4.9mH, L_b_ = 0.75mH, L_c_ = 0.88mH, i_m_ = 50A, ψ_m_ = 0.6mH after calculation.

21 $$\begin{gathered} T_{p} = \left[ {\frac{{L_{b} - L_{c} }}{2}i_{p}^{2} + Ai_{p} - \frac{A}{B}(1 - e^{{ - Bi_{p} }} )} \right]f{\prime} (\theta ) \hfill \\ T_{e,k + 1} = \sum {T_{p} } \hfill \\ i_{p,k + 1} = i_{p,k} + \frac{{T_{s} }}{{\frac{{d\psi_{p,k} }}{{di_{p,k} }}}}\left( {v_{p,k} - r_{s} i_{p,k} - \frac{{d\psi_{p,k} }}{{d\theta_{k} }}\omega_{k} } \right) \hfill \\ \theta_{k + 1} = \theta_{k} + \omega_{k} T_{s} \hfill \\ \partial \psi_{p,k} /\partial i_{p,k} = L_{b} i_{p,k} + [L_{b} i_{p} + A(1 - e^{{ - Bi_{p,k} }} ) - L_{c} i_{p,k} ]f(\theta_{k} ) \hfill \\ \end{gathered}$$

Initially, estimating the phase current and rotor position for the *k*th step from the sensors and predicting the *k*th phase torque and flux (21). The identification results of this model's parameters and the errors between it and the flux-linkage model obtained from finite element simulation is 0.735 Weber.

22 $$\begin{gathered} A = \psi_{m} - L_{b} i_{m} \hfill \\ B = (L_{d} - L_{b} )/A \hfill \\ f(\theta ) = (2N_{r}^{3} /\pi^{3} )\theta^{3} - (3N_{r}^{2} /\pi^{2} )\theta^{2} + 1 \hfill \\ \end{gathered}$$

The torque of phase p at the next sampling point, k + 1 can be predicted

23 $$T_{p,k + 1} = \left[ {\frac{{L_{b} - L_{c} }}{2}i_{p,k + 1}^{2} + Ai_{p,k + 1} - \frac{A}{B}(1 - e^{{ - Bi_{p,k + 1} }} )} \right]$$

The magnitude of flux linkage, vector, and angle can be obtained from the phase current and voltage. Then, the cost function minimizes the error and selects the optimal voltage vectors that are expressed as

24 $$\begin{gathered} \min J(V_{k} ) = (T_{p,k + 1} - T_{p,ref} )^{2} + \kappa_{1} \sum {i_{k + 1}^{2} } \hfill \\ s.t.V_{k} \in \left\{ {\left. {S_{a} ,S_{b} ,S_{c} ,\;{\text{and}}\;S_{d} } \right\}} \right. \hfill \\ \end{gathered}$$

where *J* is the cost function., and *k*_*1*_ is the weighting factor to balance the control objectives^34^. The range of weighting factor is selected from 0.50 to 0.75 to balance the control objective function.

The four-phase switching states (*S*_*a*_*, S*_*b*_*, S*_*c*_, and *S*_*d*_) are selected by optimal active large and small VVs (*V*_1_*, V*_2_, *V*_3_*,…, V*_8_). The amplitude of reference flux is assumed as constant, and reference torque is calculated using the PI controller, respectively. However, the cost function (23) shows the total torque prediction, the square of the current prediction, and flux tracking performance, respectively. This paper aims to execute the torque ripple reduction and avoid system control objective complexity. Thus, the cost function for total torque prediction is shown:

25 $$\begin{gathered} \min J(V_{k} ) = (T_{e,k + 1} - T_{e,ref} )^{2} + \kappa_{1} \sum {i_{k + 1}^{2} } \hfill \\ s.t.V_{k} \in \left\{ {\left. {S_{a} ,S_{b} ,S_{c} ,\;{\text{and}}\;S_{d} } \right\}} \right. \hfill \\ \end{gathered}$$

The steps involved in the proposed FS-MPC for the SRM drive are given as follows.

*Step 1*: The 8/6 SRM approximation FEA analysis to obtain two flux linkage curve LUT data at aligned and unaligned measurement calculated mathematical model from Eq. (21).

*Step 2*: The motor phase current (i_*k*_) and rotor position detection (ө_*k*_) from circuits.

*Step 3*: The rotor position is detected to obtain the VVs of the sector switching Table 4.

*Step 4*: The VV switching state (S_*a*_, S_*b*_, S_*c*_, and S_*d*_), phase current i_*k,*_ and rotor position өk of each phase is calculated from Eq. (21), also calculate total torque T_*e(k*+*1)*_ at the period k + 1* instant.*

*Step 5*: Calculate the cost function using (25) with eight VVs and select the minimum error VVs for the next sample instant.

## Result and discussion

### Simulation results

**Figure 12:**
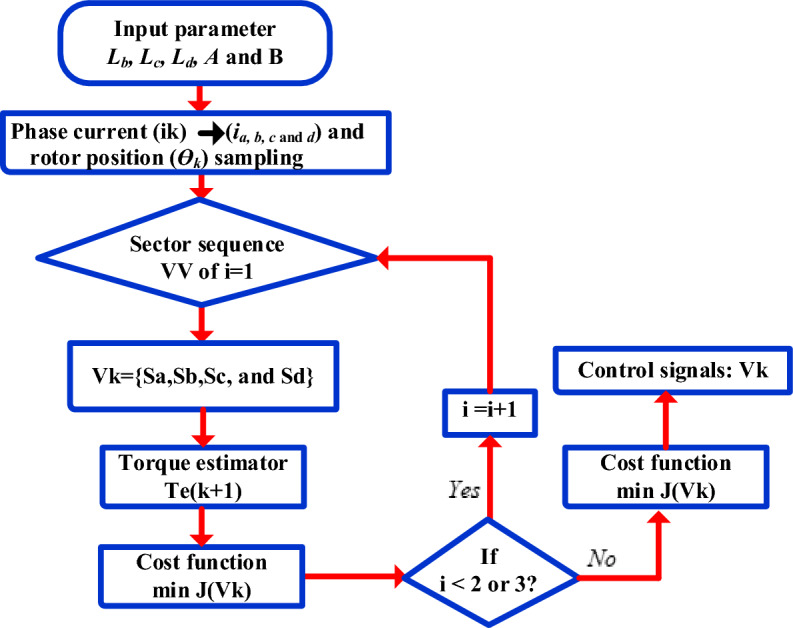
Flowchart of proposed FS-MPC.

**Figure 13:**
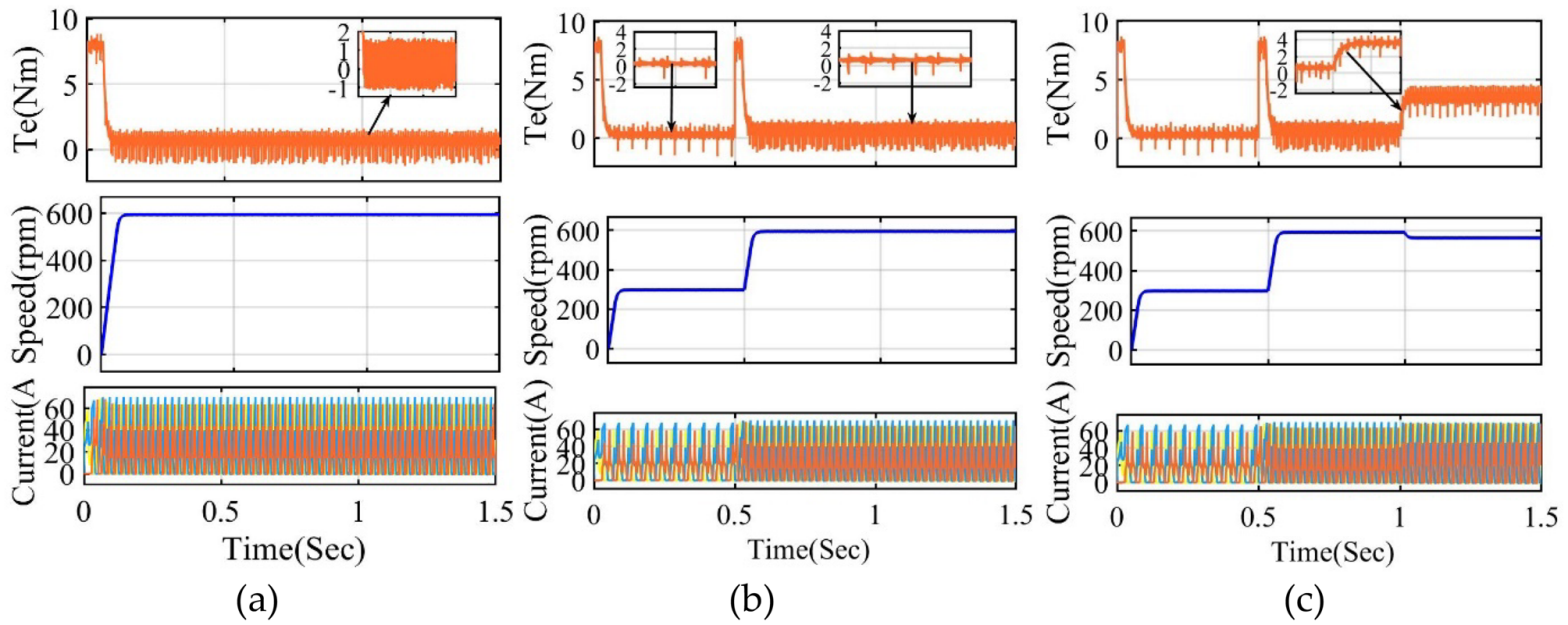
Simulation four-phase classical DTC VV response (**a**) Steady state 600 rpm torque, speed, and current (**b**) Forward step speed from 300 to 600 rpm at 0.5 s (**c**) Load response at 1 s.

The four-phase SRM model is developed, and simulation results are verified in MATLAB/Simulink. Figure 9 indicates conventional DTC techniques for four four-phase SRM drives. The DTC simulation results of instantaneous torque, speed, and phase current are presented in Fig. 12. Looking at the data from the four-phase SRM simulation results, notice that when the motor is running steadily at 600 rpm, it shows a stronger torque response. However, it experiences a higher torque ripple using Eq. (26), as shown in Fig. 13a. In dynamic forward speed response from 300 to 600 rpm at 0.5 s, the torque ripple increases compared to low speed 300 rpm, as depicted in Fig. 13b. The load torque response at 4 Nm and 1 s is shown in Fig. 13c. The response is characterized by noticeable torque ripple and spikes, indicating areas where the control method needs improvement. From static, dynamic, and load response speed conditions, it can be observed that torque ripple and spikes are high and listed in Table 4. From the results, torque ripple and flux ripples are high, and torque per ampere ratio is low, respectively. To quantify the performance of the SRM drive can be expressed as

26 $$T_{r} = \frac{{T_{M} - T_{m} }}{{T_{av} }}*100\%$$

where, *T*_*r*_ peak-to-peak torque ripple, *T*_*M*_*, T*_*m*_, and *T*_*av*_ are the instantaneous maximum, minimum and average torque, respectively.

**Figure 14:**
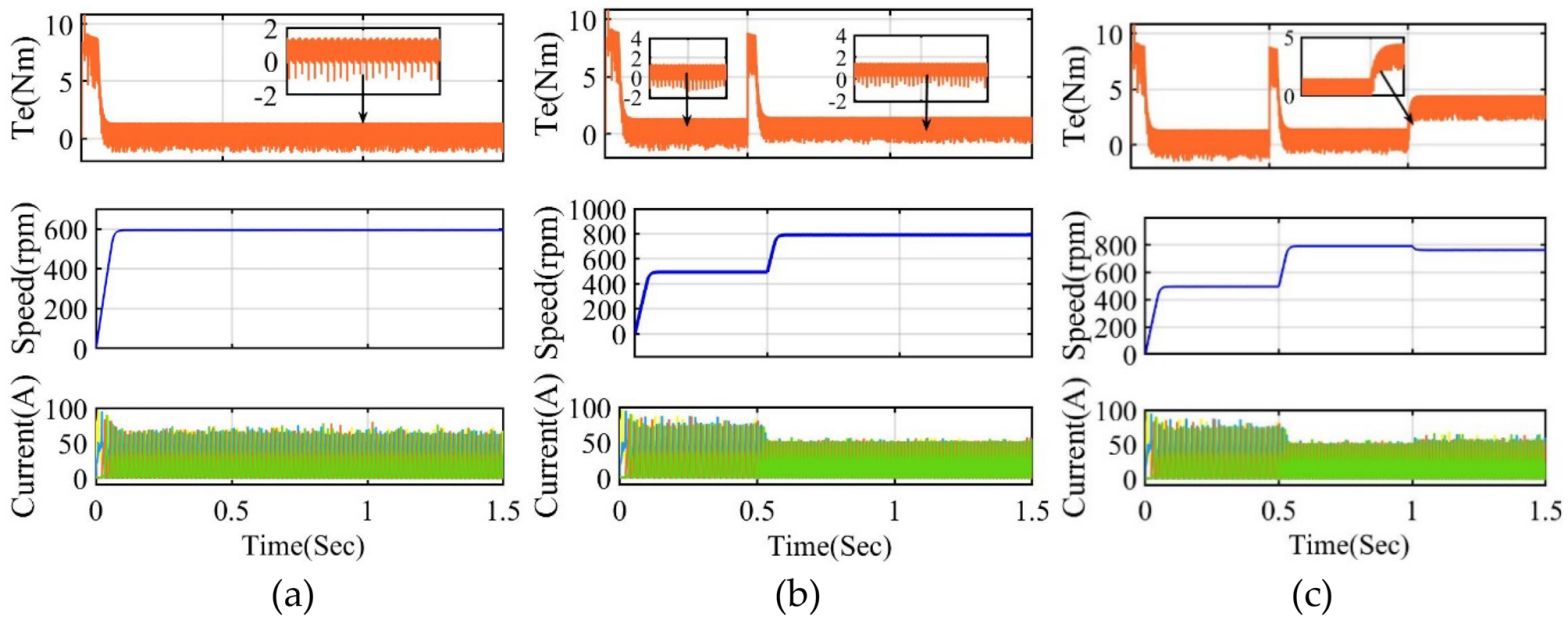
Simulation four phase proposed FS-MPC VV response torque, speed, and current (**a**) Steady state 600 rpm (**b**) Forward speed from 300 to 600 rpm at 0.5 s (**c**) Load response at 1 s.

The FS-MPC simulation results of torque, speed, and phase current are presented in Fig. 14. From the four-phase SRM results. It can be observed that a steady state speed response of 600 rpm exhibits better torque response, high torque per ampere ratio, and high torque ripple as depicted in Fig. 14a. In dynamic forward speed response from 300 to 600 rpm at 0.5 s, the torque ripple increases above 0.5 s when increasing speed response as depicted in Fig. 14b. The response depicted in Fig. 14c also shows torque ripple and spikes at 4 Nm and 1 s. where the torque ripple is less compared to classical DTC techniques. From static, dynamic, and load response speed conditions, it can be observed that torque ripple and spikes are more than 600 rpm, and below 300 rpm values are listed in Table 6. In the entire speed response, the torque ripple, and flux ripples decrease, respectively.

**Figure 15:**
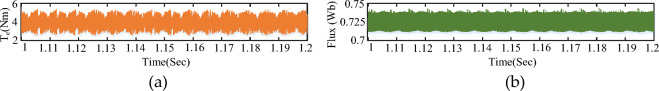
Conventional DTC torque response from 1 to 1.2 Sec period (**a**) torque ripple (**b**) flux ripple.

**Figure 16:**
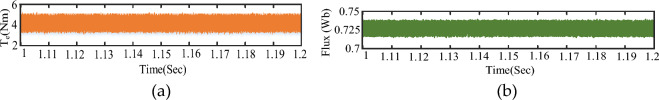
Proposed FS-MPC torque response from 1 to 1.2 Sec time period (**a**) torque ripple (**b**) flux ripple.

The parameters for the 8/6 SRM model design were initially developed using MAGNET software. Subsequently, these parameters were utilized to develop the motor and control model in MATLAB/Simulink. The simulation parameters are shown in Table 1. Figure 15a,b show the simulation results of conventional DTC with torque ripple and flux ripple. It can be seen that torque ripple and spikes are more in the speed response of 600 rpm at 1–1.2 s time period. In addition, flux ripple is also increasing within the hysteresis-loop band. The flux and torque ripple are decreased with the proper selection of the weighting factor. Therefore, the simulation results of the proposed FS-MPC with torque ripple and flux ripple as shown in Fig. 16a,b.

**Figure 17:**
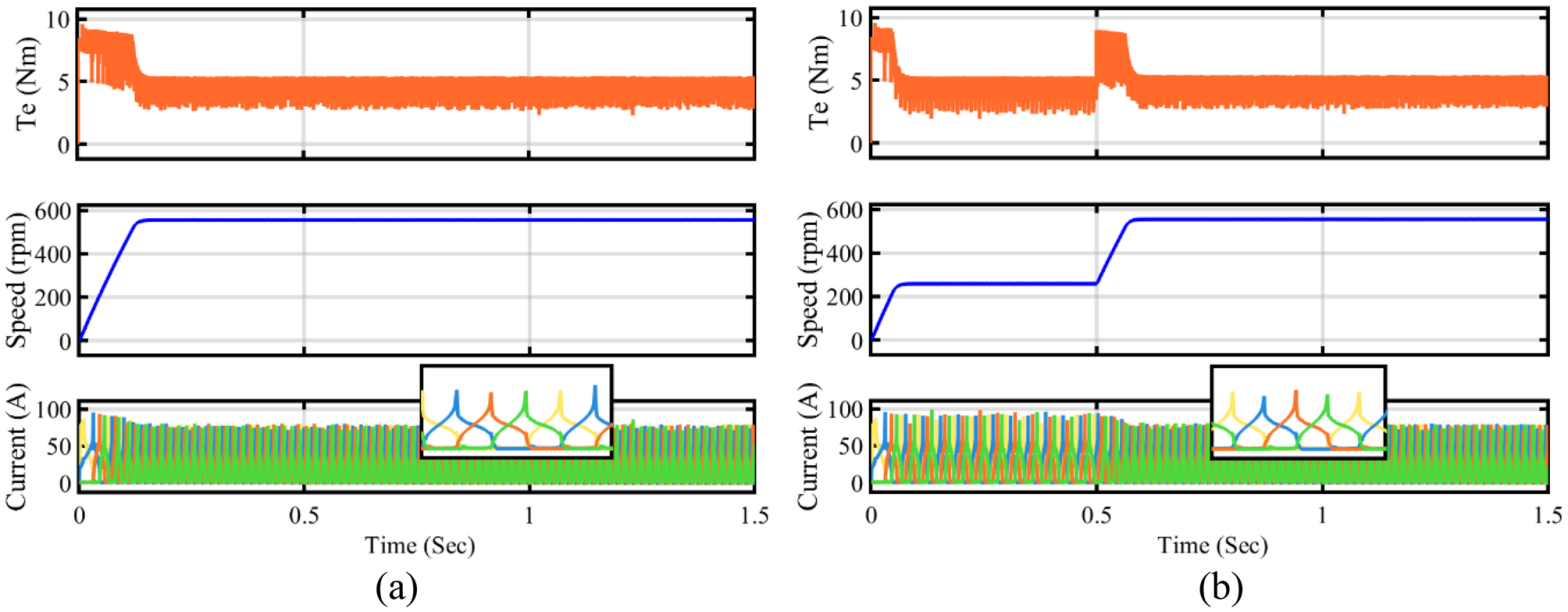
Simulation four phase DTC VV response at load torque 4 Nm (**a**) Steady state 600 rpm (**b**) Forward speed from 300 to 600 rpm at 0.5 s.

**Figure 18:**
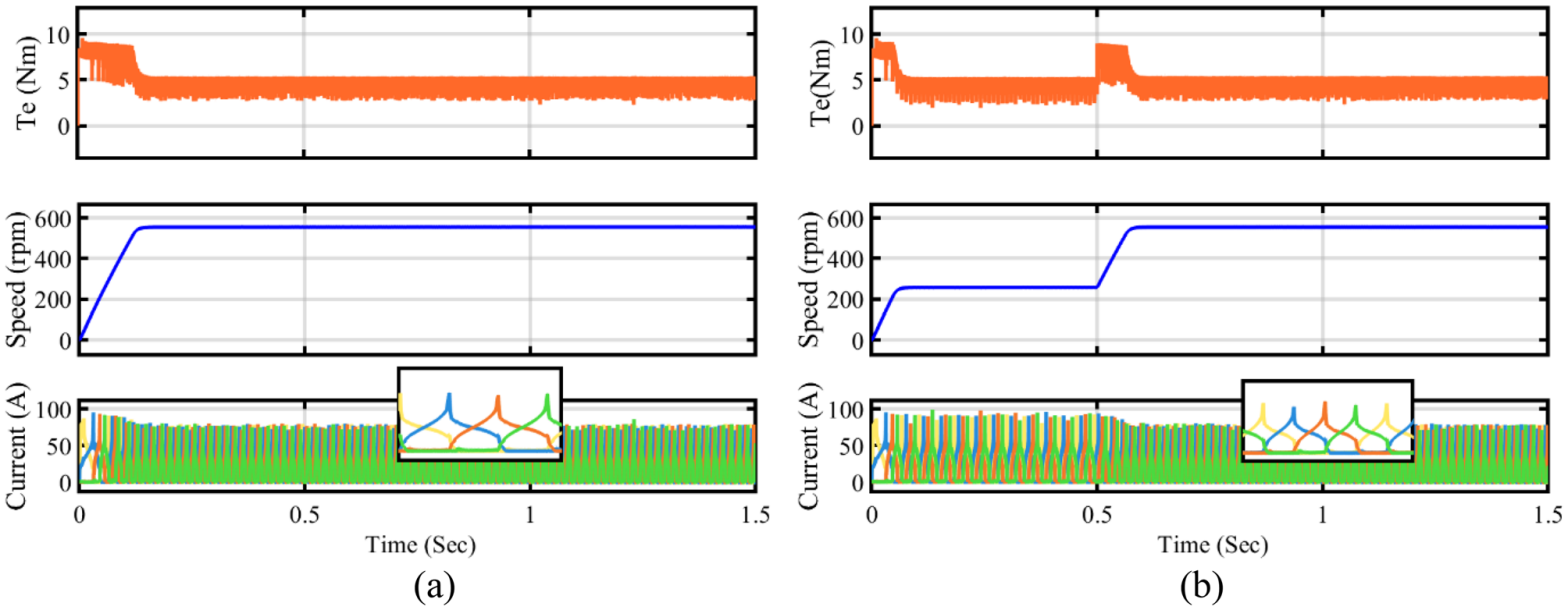
Simulation four phase proposed FS-MPC VV response at load torque 4 Nm (**a**) Steady state 600 rpm (**b**) Forward speed from 300 to 600 rpm at 0.5 s.

The speed response has different ranges from 300 to 600 rpm or above 600 rpm, and torque ripples are reduced to 2.091 Nm and 1.074 Nm. The flux and torque ripple values are obtained from the waveform and calculated using standard deviation. The proposed FS-MPC results are better than conventional DTC and MPC, such as flux and torque ripple, respectively. Increasing the load torque at 4 Nm for both operating conditions for the DTC and proposed 2.2 kW as shown in Figs. 17 and 18. Thus, the torque ripple is lesser in the proposed techniques at higher load torque applied in various speed operating conditions.

From these results, it can be verified that the proposed MPC single-cost function of four-phase SRM has better torque response, less torque ripple, and a high T/A ratio compared to conventional DTC and MPC techniques. This can be achieved by introducing new active VVs in the proposed MPC torque and flux prediction approach cost function using both high and low-speed VV execution. The average, maximum and minimum torque ripple is calculated using (25) from the simulation results for both conventional and proposed MPC. Under steady-state conditions at 300 rpm, 600 rpm, and full load, the proposed FS-MPC yields calculated torque ripple values of 27.15%, 29.54%, and 32.5%, and flux ripple values of 2.25%, 2.91%, and 3.34%, respectively.

### Experimental results

**Figure 19:**
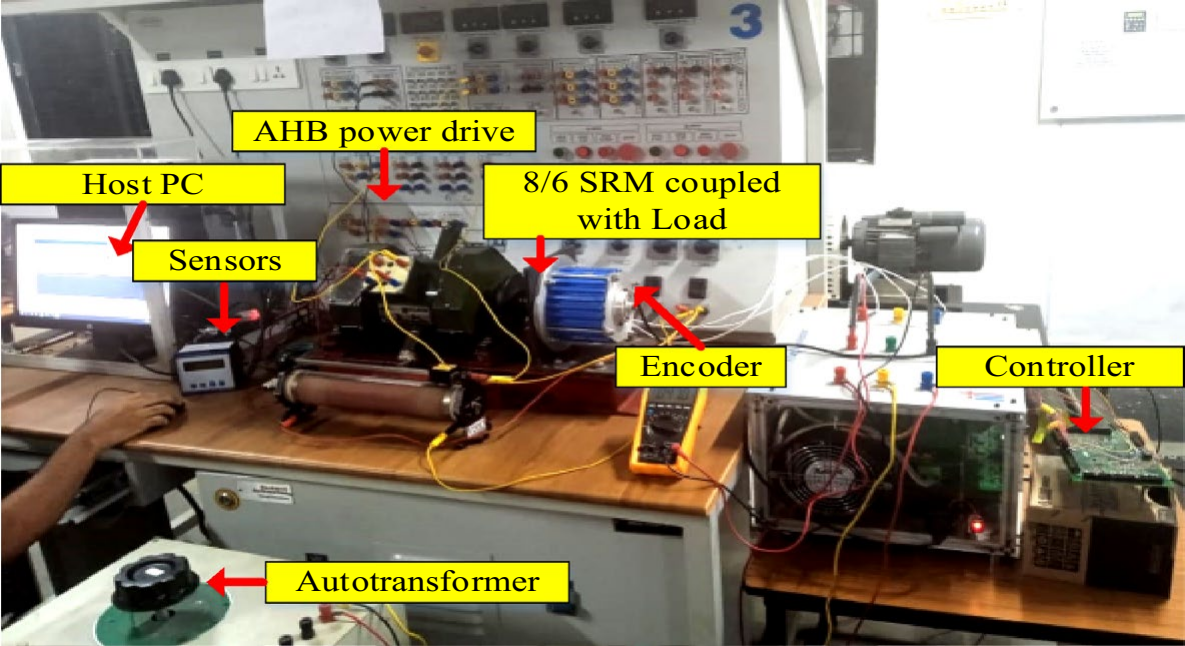
Experimental setup SRM-Drive.

The experimental setup for the SRM test is shown in Fig. 19. The control algorithm, integrating it with the motor hardware and sensors, and ensuring proper communication and synchronisation is required to implement the MPC algorithm in real-time on a suitable control platform. The AHB converter input DC link voltage is 60 V and 200 A. The hall sensor receives the position and current signals. The torque signal is detected from the torque sensor. Moreover, all these signals are transferred to the WAVECT controller (WCU300), which can operate as real-time control. The proposed FSMPC model is developed and complied with in WAVECT. Moreover, AHB converters use power switches, and high-voltage Schottky diodes are Semikron IGBT-SKM100GB12T4-20 kHz and APT2X101S20J-200 V, and 120A, respectively. The equipment used in hardware measurements is 8-channel Yokogawa DLM5000 and MSO-Tektronix MSO44 4-BW-200, Current Probe-GW INSTEK GCP300, and Voltage Probe-GW INSTEK GDP050.

**Figure 20:**
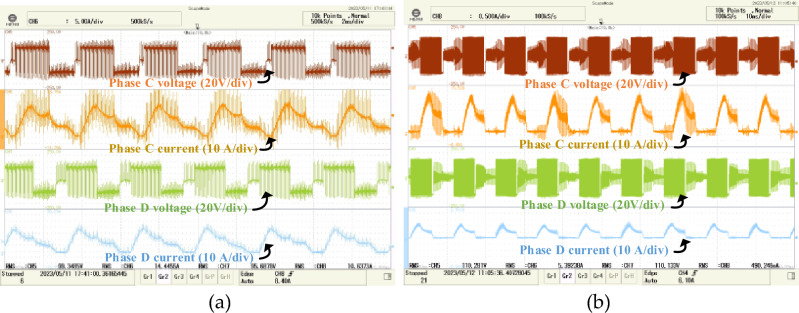
The experimental results of phase current and voltage (**a**) Conventional DTC phase A and B (**b**) Proposed FS-MPC phase A and B.

**Figure 21:**
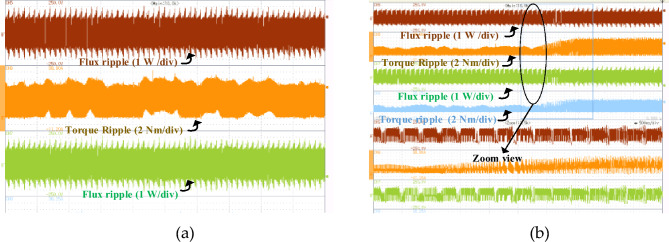
The experimental results (**a**) Proposed FS-MPC phase current and voltage (**b**) Proposed FS-MPC torque and flux ripple.

The torque and flux ripple of four-phase conventional DTC and proposed FS-MPC are measured by MSO as shown in Fig. 20. The zoom view of experimental waveform FS-MPC flux and torque ripple as shown in Fig. 21. The phase current directly influences the phase torque. Also, the flux and torque ripple of two-phase at steady state performance and dynamic load torque changes from 2 to 3 Nm are verified experimentally. The numerical value of flux and torque ripple are calculated with the help of sensor output and ripple equations. Total of 72,000 samples, with a torque sampling frequency of 10 kHz. The experimental numerical values for torque ripple were calculated using Eq. (25) and are listed in Table 6.

**Figure 22:**
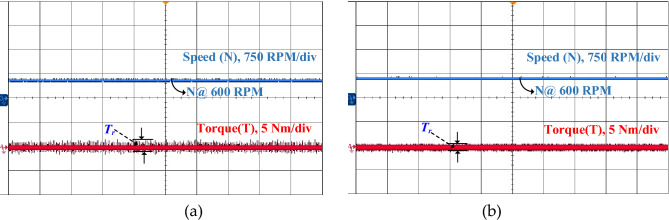
Steady state speed response 600 rpm (**a**) Conventional DTC (**b**) Proposed FS-MPC.

In a real-time four-phase SRM drive setup, the performance of steady state, dynamic and load response of conventional DTC and proposed FS-MPC techniques are measured by DSO. The motor speed and torque response of steady-state operation for a reference speed of 600 rpm for the conventional method are observed in Fig. 22a and the proposed DTC scheme steady-state response is shown in Fig. 22b. From the result, FS-MPC reduced torque ripple is calculated using the standard deviation method at 1.074 Nm. With the same speed, the flux ripple is reduced to 0.9066 Wb and torque spikes are removed in the proposed FS-MPC compared to conventional control techniques, respectively.

**Figure 23:**
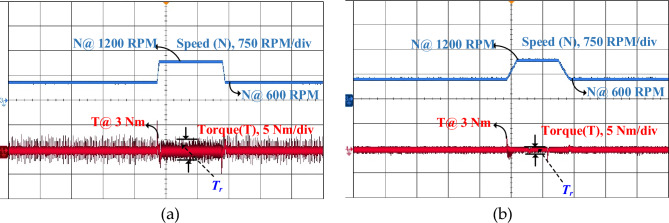
Dynamic forward acceleration speed response 1200–600 rpm (**a**) Conventional DTC (**b**) Proposed FS-MPC.

**Figure 24:**
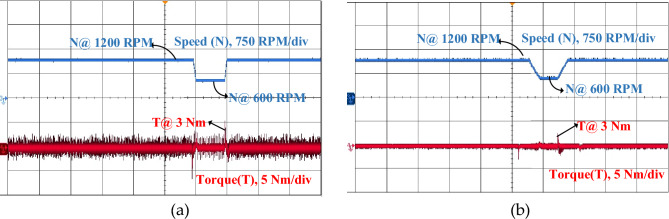
Dynamic forward deceleration speed response 600–1200 rpm (**a**) Conventional DTC (**b**) Proposed FS-MPC.

Moreover, dynamic conditions for forward acceleration and deceleration speed response are represented. Thus, dynamic forward speed response from 600 to 1200 rpm at 1 s smooth variation. From the observation, Fig. 23a produce high torque ripple and spikes in the conventional scheme. The proposed FS-MPC has smooth variation and fast dynamic response from 1200 to 600 rpm, as shown in Fig. 23b. Similarly, dynamic forward deceleration speed response from 600 to 1200 rpm at 1 s produces a minimum torque ripple of 1.074 Nm, as shown in Fig. 24a, compare to conventional control as shown in Fig. 24b. The analysis performance parameters of conventional and proposed schemes are listed in Table 5. The torque ripple and flux ripple are calculated using the standard deviation method.

**Table 5:** Comparison with other control techniques.

Controls	Torque ripple	Controller	No of VVs	Torque estimation	Modulator required	Reference
Proposed method FS-MPC	Low	Single cost function	2	Composite function	No	–
Conventional DTC	Medium	2 hysteresis loops	4	Look up table	No	^32^

*Comparison with other classical methods:* The proposed FS-MPC method, compared with the other two classical control techniques, is listed in Table 5. In^32^, Classical DTC techniques required two hysteresis loops for torque or flux. Also, it requires rotor position-current-torque characteristics to build a look-up table, which results in a high torque ripple, and calculating flux linkage is time-consuming. In^34^, the continuous control set-MPC method used in SRM drive, nonlinear behaviour of objective control estimation, prediction time, and increasing cost function make the controller complex and high cost. In^33^, MPTC evaluates 2 or 3 CVVs in each control period, reducing torque ripple effectively, it requires torque estimation from two rotor positions, making the controller complex. Therefore, the finite control set- MPC uses a single cost function objective and reduces the complexity in a single prediction time zone. The proposed scheme predictions are easy to implement, and it has low torque ripple under the speed response and load change.

**Figure 25:**
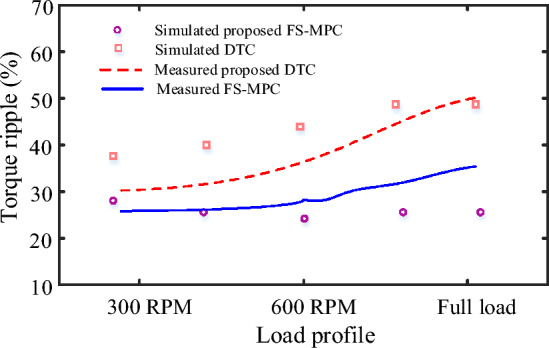
Performance comparison of torque ripple.

The results show that performance analysis on SRM's main issue of torque ripple is reduced by 27.14% at low-speed operation compared to other conventional control techniques listed in Table. 6. Additionally, torque ripple comparison of proposed with conventional DTC simulation and experimental as depicted in Fig. 25.

**Table 6:** Results comparison.

Response parameters	DTC			FS-MPC		
Speed (N) (r/min)	300	600	3 Nm	300	600	3 Nm
Average Torque (T_av_) (Nm)	1.864	3.90	4.09	1.870	3.91	4.12
Torque ripple (T_r_) (%)	39.66	40.74	43.1	27.15	29.54	32

From the overall result, the proposed FS-MPC techniques are validated with classical DTC, addressing low torque ripples in steady state speed, dynamic state (low and high speed) and load step changes in SRM drive. Hence, the proposed FS-MPC single cost function errors attain major improvement in SRM drive operation. Finally, the proposed FS-MPC is considered one of the best control strategies, showing its effectiveness in fast dynamic speed response in entire operation conditions, reducing torque and flux ripple of SRM. This strategy has robustness under different speeds and full load conditions. The results show that low torque ripple SRM drive is easy to implement in various industrial applications. Hence, the torque control scheme allows high speed and low torque ripple much needed for machine tools, vertical lathes, food mixing machinery, automotive, lifting machines, power generation, and general machinery industries. In addition, it is used for adjustable speed drive industry applications. Also, this strategy provides a low-cost controller for the SRM drive.

## Conclusion

This paper examines three distinct control techniques—DTC, MPC, and the proposed FS-MPC—in the context of torque ripple suppression utilizing a candidate voltage vector strategy. Through the selection of sector organization, voltage vector adoption, and implementation of control techniques, torque and flux are effectively managed. The proposed switching strategy demonstrates efficacy in minimizing torque ripple across various steady-state speed conditions. Simulation results validate the effectiveness of all three control techniques under dynamic speed conditions. Notably, the proposed strategy employs a predictive control objective-based single cost function to reduce system complexity, resulting in decreased torque ripples. Specifically, the results reveal a reduction of torque ripples by 27.14% in low-speed SRM drive scenarios. These findings and observations underscore the significant advantages of the proposed FS-MPC techniques for SRM drives. Given their ease of implementation and effective ripple minimization, the FS-MPC techniques hold promise for widespread application in industrial settings.

## Ethical approval

The paper is not currently being considered for publication elsewhere.
